# Intelligence

**Published:** 1809-08

**Authors:** 


### INTELLIGENCE.

The following article we select from the last Number of the Monthly Magazine. From the respectability of that Publication we presume it has not been inserted without authority.

"We have great pleasure in presenting our readers with a test of security, in regard to persons who have undergone Vaccination, and who may be made uneasy by the false and interested alarms of malignant persons. Let a patient be selected on whose arms the vaccine pustules have *regularly* advanced to the 7th, 8th, or 9th day. From one of these pustules, let the subject intended to be put to the test of security, be re-vaccinated, and at the same time, and with a portion of the same vaccine fluid, let another child, who never has had either the cow-pox or the small-pox, be also vaccinated. On the arms of the child put to this test, if it was previously previously secure, the virus will produce in a short space of time, (two or three days perhaps), an inflammation around the parts punctured, and sometimes small irregular vesicles, accompanied with itching, which commonly dies away, long before the regular pocks on the arms of the child that had not been before secured, arrive at maturity. The reason why Dr. Jenner recommends the vaccination of a child not in a doubtful state, with the one whose situation may be supposed doubtful, is to prove to a certainty, that the vaccine fluid employed, is in a state of perfection. The insertion of variolous matter by way of test, in the early periods of the vaccine practice, was adopted and recommended by Dr. Jenner; but although it did not produce the small-pox on those previously vaccinated, it sometimes occasioned very extensive and troublesome inflammation on the arms."

Dr. Wollaston has been led, by Mr. Davy's experiments on the separation and transfer of chemical agents by means of the Voltaic apparatus, to imagine it probable that animal secretions are effected by the agency of a similar electric power. In this opinion he is supported by the following experiment.—He took a piece of glass tube about three quarters of an inch in diameter, and nearly two inches long, open at both ends, and covered one of them with a piece of clean bladder. Into this little vessel he poured some water, in which had been dissolved $\frac{1}{240}$ of its weight of salt; and after placing it upon a shilling, with the bladder slightly moistened externally, he bent a wire of zinc, so that while one extremity rested on the shilling, the other might be immersed about an inch in the water. By successive examination of the external surface of the bladder, he found that even this feeble power occasioned soda to be separated from the water, and to transude through the substance of the bladder. The presence of alkali was discernible by the application of reddened litmus paper, after two or three minutes, and was generally manifest even by the test of tumeric before five minutes had expired. This experiment tends to confirm the conjecture that similar agents may be instrumental in effecting the various animal secretions which have not yet been otherwise explained. The qualities of each secreted fluid may hereafter instruct us in the species of electricity that prevails in each organ of the body.

M. Boullay, of Paris, has invented an improved process for preparing sulphuric ether, which he describes as follows:—To a large tubulated glass retort placed on a sand heat, I adapted a glass worm immersed in a vessel of cold water. The extremity of the worm was inserted in the neck of a large bottle, between which and a second bottle filled with water, a communication was established by means of a siphon. Into the retort I introduced 22lbs. of sulphuric acid, concentrated to 66°. In the tubulure was inserted a particular kind of funnel with two cocks, so that its pipe descended nearly to the bottom of the retort, passing through the sulphuric acid: 22lbs. of alcohol at 36° of Beaumé's areometer were then poured in quickly, being conveyed through the acid by means means of the funnel. The mixture was very well effected; the distillation was kept up by means of a fire under the retort, and as soon as about 4lbs. had passed over, 22lbs. of fresh alcohol, at 40° were introduced, drop by drop, regulating the quantity as nearly as possible by what passed over into the receiver. The process was continued so as to obtain 33lbs. of a fine white limpid product, of the most agreeable etherial smell and taste, containing no traces of sulphurous acid, and yielding, when rectified on a water bath, 17½ lbs. of pure ether, with some alcohol of an etherial smell, well adapted for future processes. The residuum consisted of nearly the whole of the sulphuric acid employed, some alcohol, water, and probably a certain quantity of ether completely formed; and might be used as sulphuric acid where the alcohol could do no harm, as for instance in forming different salts.

The following account of the recent eruption of Mount Etna, in Sicily, has been transmitted by an eye-witness:—On Tuesday, the 27th of March, the inhabitants of the neighbourhood of the mountain were frightened by repeated and violent shocks of an earthquake; these were soon followed by immense volumes of ashes and other volcanic matter, thrown up from the mouth of the crater to a great height, so as to darken the sky, and to fill all the environs with ashes, Messina not excepted, which lasted five hours and a half. This, however, was only a prelude. On the 28th, towards evening, the fiery matter forced its way through two apertures below the crater, and was thrown up to a prodigious height. The flames and red-hot stones afforded a most sublime spectacle. On the 29th, and following days, upwards of twenty different apertures were formed in different parts of the mountain, from all which the eruptions of fire and stones in all directions were tremendous. Streams of red-hot lava ran down profusely, and in their descent destroyed every thing at a touch. A fine chesnut wood, several extensive olive plantations and vineyards, became instantly a prey to the liquid element. Upwards of twelve square miles are inundated by the fiery torrent. Of the many apertures, two in particular continued vomiting fire and lava for several days, and caused the greatest damage. No lives have been lost, although the village of Lingua Glossa was nearly destroyed. A stream of lava approached it within two miles, and would have reached it in its descent, had it not stopped of its own accord. At first the lava travelled at the rate of one yard per minute; about a week ago its velocity was reduced to one or two feet per hour. It is about six miles down the mountain; some of its channels are half a mile in breadth, and 60 to 80 feet deep. Several new mountains have been formed on the base of the old one. No exact estimate has yet been formed of the damage occasioned by the eruption; but the extent and value of the landed property destroyed by it must be very great, and a number of families, who solely subsisted on the produce of their fields, have been reduced to a state of absolute beggary.

Dr.

Dr. Ramsbotham will commence his Lectures on the Science and Practice of Midwifery, on Monday October the 2d, at his house, No. 9, Old Jewry.—These Lectures are illustrated by anatomical preparations, and casts from his valuable Museum, to which the Pupils will have the advantage of free access for individual improvement.—Two gentlemen may be accomodated as house Pupils, who will receive every domestic attention, as well as derive considerable professional advantages.

Mr. J. Wilson, surgeon, late of Guy's Hospital, will speedily publish Pharmacopæia Chirurgia, or Formulæ of the different Hospitals.

